# Can High Risk Patients With Cholangitis be Identified?

**DOI:** 10.1155/1990/36580

**Published:** 1990

**Authors:** John Ham

**Affiliations:** Department of Surgery Prince of Wales Hospital High Street Randwlck, NSW 2031 Australia


*HPB Surgery*, 1990, Vol. 2, pp. 215–223
Reprints available directly from the publisher
Photocopying permitted by license only

© Harwood Academic Publishers GmbH
Printed in the United Kingdom

### HPB INTERNATIONAL

**EDITORIAL & ABSTRACTING SERVICE**

JOHN TERBLANCHE, EDITOR
Department of Surgery,
Medical School · Observatory 7925
Cape Town · South Africa
Telephone: (021) 47-1250 Ext. 229
Telefax (021) 47-8955

### CAN HIGH RISK PATIENTS WITH CHOLANGITIS BE IDENTIFIED?

### ABSTRACT

*Gigot JF, Leese T, Dereme T, Coutinho J, Castaing D and Bismuth H. Acute Cholangitis : Multivariate Analysis of Risk Factors. Ann Surg 1989; 209 No. 4:435–438.*

**In order to identify risk factors in patients with acute cholangitis, 140 clinical, biochemical, etiologic and pathologic variables of 449 attacks of acute cholangitis seen in one center over a 20-year period were analyzed. Simple regression revealed 24 factors with prognostic significance but multivariate analysis detected only seven factors with independent significance in predicting mortality (acute renal failure, cholangitis associated with liver abscesses or liver cirrhosis, cholangitis secondary to high malignant biliary strictures or after percutaneous transhepatic cholangiography, female gender, and age). When the presence of each of these factors is weighted proportional to its coefficient of regression, patients with cholangitis could be scored on a scale of 0–27. A score of seven was clinically the most useful cut off – 388 attacks of cholangitis associated with a score of $<$ 7 had a mortality rate of only 1.8%, whereas 61 attacks associated with a score $\geq$ 7 had a mortality rate of 49%,. The value of this scoring system needs to be confirmed in prospective studies, but it may prove useful, for example, in selecting a group of high-risk patients for urgent biliary decompression in an attempt to reduce the mortality associated with this pathology.**

**PAPER DISCUSSION**

KEYWORDS: Biliary tract, cholangitis, multivariate analysis, risk factors

Acute cholangitis is a syndrome of variable severity. The majority of patients respond to antibiotics and conservative therapy, and may then have an elective or semi-elective procedure to treat their biliary obstruction. A minority with severe sepsis do not respond and require urgent biliary decompression. It is often assumed that this latter group of patients has suppurative cholangitis but, as Boey & Way[1] have pointed out, the correlation between biliary suppuration and clinical manifestations is inexact – "some patients with severe sepsis do not have pus in the bile duct, and a few patients with suppurative bile are only moderately ill." In addition, a retrospective comparison of patients with suppurative and non-suppurative cholangitis did not demonstrate any significant differences in terms of histological data, physical findings or preoperative laboratory values.[2] Other authors [3,4] have emphasized the importance of early biliary decompression in patients who do not rapidly respond to conservative treatment; however the definitions of "rapid response" and "early decompression" are imprecise.

The stated purpose of this study was the identification of factors useful in predicting mortality in acute cholangitis at an early stage of the disease process, with a view to identifying a high risk group of patients who might be selected for urgent decompression of the biliary tree. The aim is therefore similar to that which prompted the development of prognostic scoring systems in acute pancreatitis.

The study is important in that the authors have used multivariate analysis to reduce the number of identified risk factors to those which had independent significance in predicting mortality. However, it should be appreciated (as the authors point out) that the study comes from a specialist hepatobiliary unit, and that it analyses patients seen over a 20 year period.

The data is based on 449 attacks of acute cholangitis in 412 patients. Sixty-one per cent of the patients had had biliary tract surgery previously, and 10% had cirrhosis (4.9% non-biliary and 5.1% biliary). The cholangitis was related to stones in 48%, benign biliary strictures in 28% and malignant strictures in 11%. Presumably, many patients in the latter two groups had recurrent obstruction, and such patients present particularly difficult problems. Despite the unusual nature of the patient population, pus in the biliary tract (suppurative cholangitis) was noted in only 6.7% of attacks and liver abscess in 5.9%. Reynold's pentad (fever, jaundice, abdominal pain, shock and abnormal mental status) was present in 3.5% of attacks; it was significantly associated with mortality.

The importance of the initial response to antibiotic therapy is stressed in the text. Those attacks that responded immediately were associated with a mortality of 1.5%, whereas those that did not had a mortality of 62%. However the timing of any biliary decompression (surgical or non-surgical) in the latter group is not indicated. A recent paper[5] from Hong Kong is relevant to this point, even though it deals with patients with recurrent pyogenic cholangitis. One hundred and five patients with acute calculous cholangitis who did not respond to conservative management (there were 245 patients who did respond) had urgent endoscopic drainage of the billiary tract at a mean of 1.5 days (range 6 hours to 8 days) after admission. The overall mortality was 4.7%.

Despite these criticisms, the data from this study provides the basis for the assessment of a predictive scoring system, both in patients treated by modern techniques and in a less highly selected group of patients, and the authors indicate that such studies are planned.

Professor John Ham
Department of Surgery
Prince of Wales Hospital
High Street
RANDWICK, NSW 2031
AUSTRALIA

### REFERENCES

1. Boey, J. H. and Way, L. W. (1980) Acute cholangitis. *Ann. Surg.*, **191**, 264–270.
2. O'Connor, M. J., Schwartz, M. L., McQuarrie, D. G. and Sumner, H. W. (1982) Acute bacterial cholangitis. *Arch. Surg.*, **117**, 437–441.
3. Thompson, J. E., Tompkins, R. K. and Longmire, W. P. (1982). Factors in management of acute cholangitis. *Ann. Surg.*, **195**, 137–145.
4. Leese, T., Neoptolemos, J. P., Baker, A. R. and Carr-Locke, D. L. (1986). Management of acute cholangitis and the impact of endoscopic sphincterotomy. *Br. J. Surg.*, **73**, 988–992.
5. Leung, J. W. C., Chung, S. C. S., Sung, J. J. Y., Banez, V. P. and Li, A. K. C. (1989) *Lancet*, **i**, 1307–1309.

### THE CURRENT PLACE OF SHOCK-WAVE LITHOTRIPSY FOR BILE DUCT STONES

### ABSTRACT

*Sauerbruch T, Stern M. (1989) Fragmentation of bile duct stones by extracorporeal shock waves: A new approach to biliary calculi after failure of routine endoscopic measures. Gastroenterology, Vol. 96: 146–152*

**A prospective uncontrolled multicenter trial was performed on 113 patients with bile duct stones in whom routine endoscopic approaches for removal of the calculi had failed. These represented 8.3% of the patients referred to the participating centers for endoscopic extraction of the stones. Extracorporeal shock-wave lithotripsy using the Dornier kidney lithotripter achieved stone disintegration in 103 patients (91%). Complete stone clearance from the bile ducts was obtained in 97 patients (86%) after a median of 4 days following extracorporeal shock-wave lithotripsy. Adverse effects, mostly mild, occurred in 36% of the patients. A 30-day mortality rate of 0.9% (in-hospital mortality rate = 1.8%) of this high-risk group with a mean age of 72 yr and a cholangitis rate of 26%, compared favorably with the data given for open surgery. We therefore consider extracorporeal shock-wave lithotripsy a useful method for the treatment of bile duct stones not amenable to routine endoscopic measures**

